# Automated detection of age-related macular degeneration in color fundus photography: a systematic review

**DOI:** 10.1016/j.survophthal.2019.02.003

**Published:** 2019

**Authors:** Emma Pead, Roly Megaw, James Cameron, Alan Fleming, Baljean Dhillon, Emanuele Trucco, Thomas MacGillivray

**Affiliations:** a VAMPIRE Project, Centre for Clinical Brain Sciences, The University of Edinburgh, Edinburgh, Scotland; c MRC Human Genetics Unit, The University of Edinburgh, Edinburgh, Scotland; b Princess Alexandra Eye Pavilion, Edinburgh, Scotland; d Optos plc, Queensferry House, Carnegie Campus, Dunfermline; e VAMPIRE Project, Computing (School of Science and Engineering), University of Dundee, UK

**Keywords:** age-related macular degeneration, age-related disorders, artificial intelligence, machine learning, deep learning

## Abstract

The rising prevalence of age-related eye diseases, particularly age-related macular degeneration, places an ever-increasing burden on health care providers. As new treatments emerge, it is necessary to develop methods for reliably assessing patients' disease status and stratifying risk of progression. The presence of drusen in the retina represents a key early feature in which size, number, and morphology are thought to correlate significantly with the risk of progression to sight-threatening age-related macular degeneration. Manual labeling of drusen on color fundus photographs by a human is labor intensive and is where automatic computerized detection would appreciably aid patient care. We review and evaluate current artificial intelligence methods and developments for the automated detection of drusen in the context of age-related macular degeneration.

## 1 Introduction

With longer life expectancy, age-related disorders are increasing the burden placed on health care providers. In particular, age-related macular degeneration (ARMD) is one of the major causes of vision loss in the elderly.^28^, ^30^ ARMD currently affects 6 million people in the UK alone^28^ and was estimated to have cost the country's economy £155 million in 2011.^49^ By 2040, the number of people affected globally by the disease is projected to be 288 million.^58^

The earliest phase of ARMD is typically observed as the presence of (asymptomatic) macular drusen, often incidentally found on examination or fundus imaging. Drusen are small deposits of predominantly lipid, acellular debris that accumulate between the retinal pigment epithelium and Bruch's membrane. Although the presence of small drusen is not itself diagnostic of ARMD, as drusen frequently occur in normal aging, increasing number and size of drusen increase the risk of progression to visually symptomatic ARMD. Later signs of ARMD, such as pigmentary changes of the retinal pigment epithelium that occur before the development of geographic atrophy (so-called dry ARMD) and exudative abnormalities (so-called wet ARMD) enable more established gradings^5^, ^3^, ^33^ and classification of ARMD.^2^, ^28^, ^32^, ^34^

Drusen appear as clusters of white or yellow spots in color fundus photographs and broadly exist as two main types, hard and soft. Hard drusen are round, small, discrete lesions with defined edges, whereas soft drusen are less defined and often confluent. Drusen are rarely homogenous in their composition. Because of their yellow color and brightness on color fundus photographs, drusen are distinguishable by the human eye, but computer algorithms to automatically detect them need to be robust to the presence of other similarly brightly appearing pathology such as hard exudates. Indistinct borders for drusen appearing in color fundus photographs are challenging for conventional image-processing techniques such as edge detection and morphological filtering and have been discussed in detail in an earlier review.^15^ To the best of our knowledge, no reviews cover recent developments, involving the application of artificial intelligence (AI) and deep learning (DL) techniques.

**Fig. 1:**
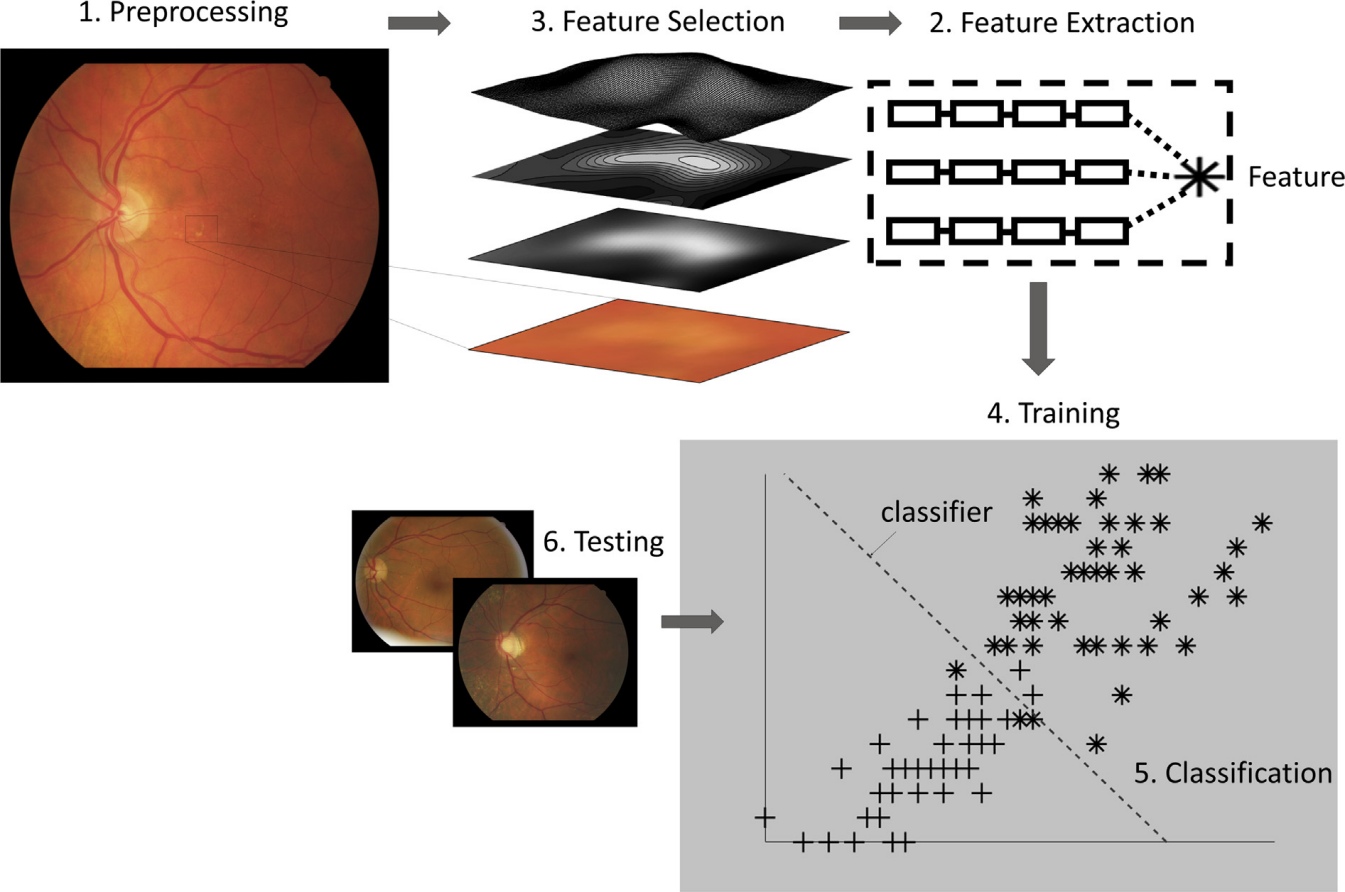
Illustration of supervised machine learning pipeline. 1) Image preprocessing is performed to reduce noise and enhance image features. 2) Features such as measures of entropy, energy, color and texture of image intensities, and spatial or geometric properties are extracted. 3) Features are grouped as numerical vectors (forming the image representation) and often undergo a selection process to decide which features best represent the image. 4) Training phase builds a model that tries to separate the data into the target, distinct classes. 5) The classifier—the mathematical function—that implements classification and defines the classes. 6) Testing is performed by classifying unseen data belonging to know classes.

AI is a long-standing field of computer science that aims to simulate human intelligence by perceiving its environment and taking appropriate action to achieve a set of goals, one of which is decision-making. Machine learning (ML) is an approach to AI, partially inspired by how humans learn.^37^ Learning is achieved through examples. If a child is presented with a new object, they will use features such as color, shape, and texture so that when they observe the object again they will use what they have learned to identify or categorize it as something they have previously seen. Similarly, many ML classification algorithms use features from training examples to discover or confirm patterns that categorize subsets. When new, unseen data are presented, the algorithm can classify which category they belong to (Fig. 1). These features can be learned by either training from previous examples (i.e., supervised learning) or discovered by the algorithm (i.e., unsupervised learning).

DL is a subset of ML that is gaining prominence for medical imaging^38^, ^45^ and ophthalmology^14^ because of increasing reports of high performance for clinical classification and decision-making. DL is based on neural networks, a class of algorithms inspired by the human brain. In a neural network, the neurons are organized in layers and implement simple operations on the input data or from the output of previous layers. In a deep neural network, the number of layers is much higher than that in conventional neural networks (indicatively 10 or more as opposed to 2-3). The connections between the layers are assigned values, called weights, representing connection strengths. Learning the weights is the objective of the training process. Training and testing a deep neural network require large amounts of labeled data (i.e., known classes).

**Fig. 2:**
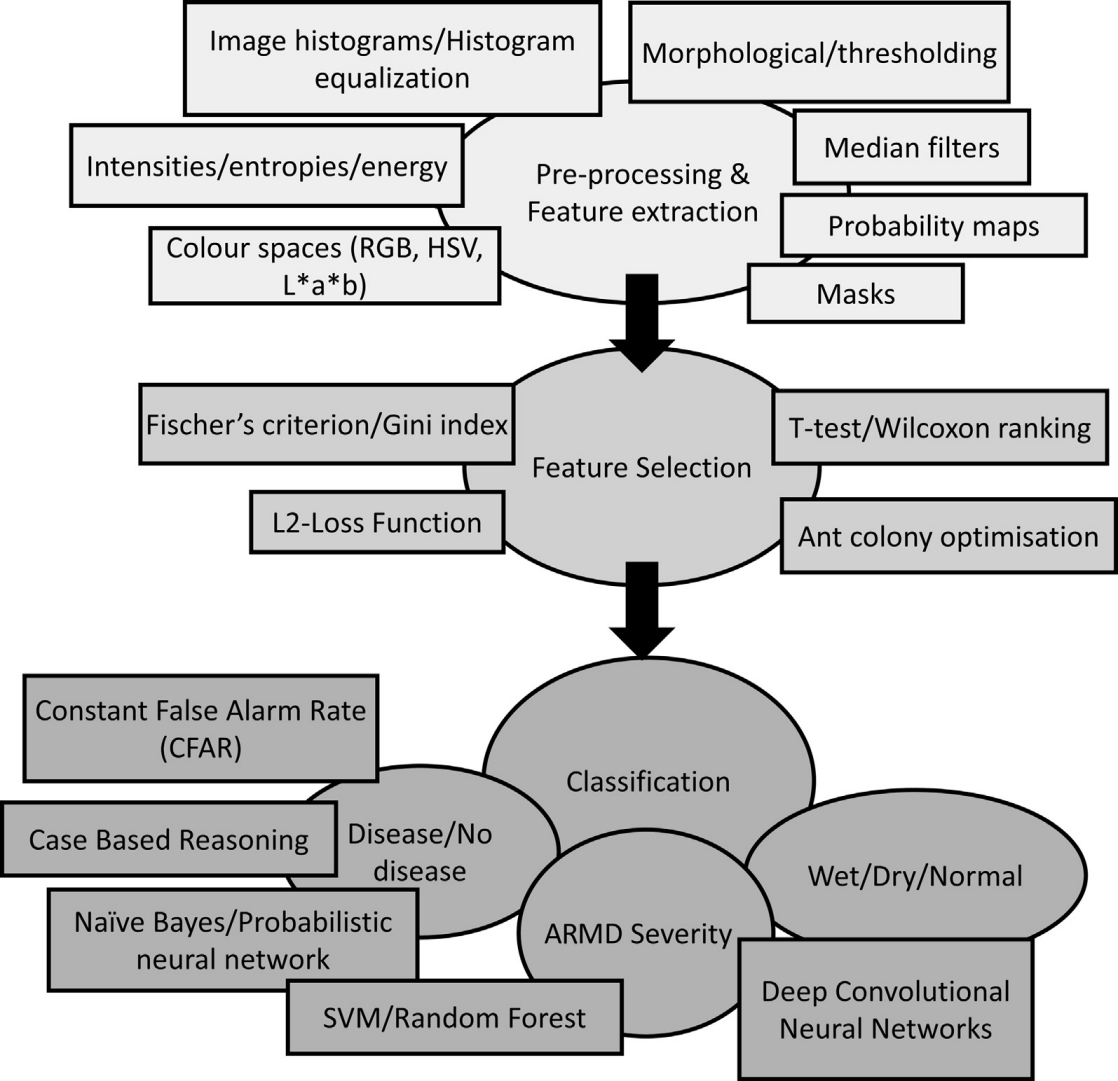
An overview of the ML methods in discussion and where they are applied at each stage. Deep Convolutional Neural Networks is a DL technique. ARMD, age-related macular degeneration; DL, deep learning; HSV, hue, saturation, value; ML, machine learning; RGB, red, green, blue; SVM, support-vector machine.

In this review, we report and evaluate current AI strategies and developments for the automated detection of drusen in the context of ARMD (Fig. 2). Although some recent work has begun to explore the potential for automated drusen detection by optical coherence tomography, with varied methods and mixed results,^10^, ^14^, ^27^, ^50^, ^56^, ^60^ the focus of this review is on color fundus imaging of the retina.

## 2 Methods

### 2.1 Inclusion and exclusion criteria

We aimed to include all published studies applying AI to automatic drusen detection in color fundus photographs. Inclusion criteria were (1) original study, (2) those written in English, and (3) those that had validation by performance against at least one manual grader. The following studies were excluded: (1) reviews; (2) nonhuman research; (3) non-English language studies; (4) studies that involved methods other than color fundus photography (e.g., optical coherence tomography); (5) studies that did not feature robust validation, as outlined in the following paragraph.

Validation is the process of showing quantitatively that an algorithm performs correctly through comparison of its output to a reference standard, for example, manual grading of images by experts.^57^ Any article that did not include validation was excluded. The performance of an algorithm is typically measured using criteria such as accuracy, sensitivity, specificity, and area under receiver operating characteristic.^24^ Another important aspect is the size of the data set: the image set, algorithm tested, must be sufficiently large to be representative of the target population and to be suitable for the number of neural network parameters to be trained. AI methods are not immune to small sample size effects that can contaminate the evaluation of a proposed system. For instance, color fundus photographs can differ in appearance between patients, and disease manifestations are also of a varying nature. Considering this, articles that mentioned validation of less than 50 images were excluded.

### 2.2 Data extraction

For all identified studies, an independent reviewer (E.P.) screened the titles and abstracts. Irrelevant and duplicate articles were removed, and the remaining articles were assessed for agreement with the inclusion and exclusion criteria by full-text review. Data extracted from studies at this stage included title, year of publication, authors, study aim, study type, number of images (training and test), diagnostic criteria, participant selection criteria, method of fundus imaging, algorithm, performance metric(s) results, and conclusions. The most recent articles were hand searched following the same strategy, filtered for the current year (i.e., 2018), and subjected to the same inclusion criteria. A similar strategy was followed for articles cited within the bibliographies of the results.

## 3 Results

A total of 2236 articles were identified in the initial search performed in 2017. After filtering for ARMD, 1318 articles were excluded, such as those featuring diabetic retinopathy (n = 42) and glaucoma (n = 42). From the remaining 918 articles, 834 were excluded because they did not use color fundus photographs (n = 18), did not use imaging (n = 770), or were not reviews (n = 34). Seventy-three articles did not meet the selection criteria, such as articles not reporting performance (n = 9) or featuring software optimization (n = 3), hardware reports (n = 2), or fewer than 50 images for validation (n = 12). At the end, 8 articles met all inclusion criteria. One additional article was included after searching bibliographies, and 5 articles were found by hand searching for this current year (2018). The resulting 14 articles were considered in this review. They all used ML and DL techniques for drusen detection in color fundus photographs.

### 3.1 Study designs and populations

The 14 studies involve 4 publicly available data sets (i.e., automatic retinal image analysis,^62^ STructured Analysis of the REtina,^26^ Age Related Eye Disease Study [AREDS],^2^ and RetinaGallery^12^), 3 private data sets and 1 sourced from a telemedicine platform and a cohort from an independent study.^6^ Some studies contained overlapping report analyses on the same data sets, but used different methods. Four articles aimed to achieve disease or no-disease classification. Six articles aimed to classify ARMD severities according to AREDS^2^ or in-house grading criteria (Cologne Image Reading Center and Laboratory [CIRCLE]). Two articles aimed to classify dry ARMD vs. normal images and 1 wet ARMD vs. dry ARMD or normal (Table 1).

**Table 1:** Included articles using AI methods for automated detection of ARMD

Reference	Data set	Fundus camera (resolution)	Preprocessing	Feature	Output
Hijazi et al 2010^25^	144 (ARIA)	Not reported	CLAHE; retinal vessels segmented by thresholding and OD segmented using intensity peaks of image (identified by sliding window)	RGB and Hue Saturation Intensity (HSI) histogram of each image conceptualized to set of curves (time series)	Disease/no disease
Burlina et al 2011^7^	66 (private)	Zeiss FF4 40° FOV (pupils dilated); images resized to 1000 × 1000	Pyramid decomposition of green channel for regions of high gradient magnitude to create logical masks for training and testing. Areas of high gradient magnitude indicate artifacts and vessels where low gradient magnitude indicate normal retinal tissue	Intensity, color, and gradient features of background (normal retina) and candidate abnormal areas	Disease/no disease
Zheng et al 2012^61^	101 (ARIA); 97 (STARE)	TOPCON TRV-50 fundus camera 35° field of view (700 × 605)	Mask of whole image to capture circular fundus ROI. Color normalization and uneven illumination is applied. CLAHE to enhance contrast. Blood vessels identified using wavelet features.	Image represented as quadtree, separated by their homogeny, defined by similar pixel values. Image mining algorithm returns features	Disease/no disease
Kankanaballi et al 2013^31^	2772 (NIH AREDS)	Not reported	Green channel smoothed by large median filter. Median filtered image subtracted from original green channel and the result multiplied to increase contrast	SIFT/SURF features of L*a*b color channel	ARMD severity
Grivensen et al 2013^20^	407 (EUGENDA)	TOPCON TRC 501 × 50° field of view; Canon CR-DGi (nonmydriatic) 45° field of view	Drusen manually outlined	Each pixel in image assigned probability that it belongs to drusen candidate. Boundary of the candidate extracted using intensity and contrast characteristics	ARMD severity
Mookiah et al 2014^43^	161 (ARIA); 83 (STARE); 540 (KMC)	Carl Zeiss Meditec fundus camera 50° field of view (748 x 576); TOPCON TRV-50 fundus camera 35° field of view (700 x 605); TOPCON non-mydriatic retinal camera (TRC-NW200) (480 x 364)	CLAHE	Entropy features: Shannon, Kapur, Renyi, Yager; higher order spectra (HOS)	Wet/dry/no disease
Mookiah et al 2014^42^	540 (KMC)	TOPCON nonmydriatic retinal camera (TRC-NW200) (480 x 364)	CLAHE	Features for whole image obtained by discrete wavelet transform (DWT) decomposition. Linear features extracted from wavelet coefficients (mean, variance, skewness, kurtosis, Shannon entropy, Renyi entropy, Kapur entropy, relative energy, relative entropy, entropy, Gini index).	Wet/dry/no disease
Burlina et al 2016^8^	5500 (NIH AREDS)	Not reported	Resizing and cropping images to conform to the expected OverFeat input network	SURF, SIFT, wavelet features	ARMD severity
Phan et al 2016^47^	279 (telemedicine platform)	Zeiss, DRS, Topcon models 45° FOV (1400, 2,200,3240 pixels along diameter of image)	Preprocessing from^31^	Color histograms (RGB, L*a*b color spaces) Texture: local binary patterns, histogram of oriented gradients (HOG), SURF	ARMD severity
Acharya et al 2017^1^	945 (KMC)	Zeiss FF450 plus mydriatic fundus camera (resized to 480 × 360 from 2588 × 1958)	CLAHE	Pyramid of histograms of orientated hradients (PHOG) to describe the shape and pattern. Features from descriptor—energy: uniformity of image; entropy features: approximate, fuzzy, Kolmogorov-Sinai, modified multiscale, permutation, Renyi, sample, Shannon, Tsallis, and wavelet Nonlinear features: fractal dimension (D), Hjorth (activity, complexity, mobility parameters), Kolmogorov complexity, largest Lyapunov exponent, Lempel Ziv complexity, relative qualitative analysis (parameters entropy, transitivity, trapping time, recurrence of the first type and second type, longest vertical line), entropy, determinism, laminarity, maximal diagonal line length, averaged diagonal line length, recurrence rate, recurrence time of RQA parameters	Wet/dry/no disease
Burlina et al 2017^9^	5664 (NIH AREDS)	Not reported	Resizing and cropping images to conform to expected OverFeat input network	OverFeat (OF) universal features	ARMD severity
Garcia-Floriano et al 2017^18^	397 (STARE); 70 (RetinaGallery)	Not reported	OD located using.^17^ Green channel.	Hu moments were used to describe each object as a measurable quantity calculated from the shape of a set of points	Disease/no disease
Tan et al 2018^55^	1110 (KMC)	Zeiss FF450 plus mydriatic fundus camera (2588 x 1958)	Image rescaled to 180 x 180 to conform to network input dimensions	Features learned through neural network	Disease/no disease
Grassman et al 2018^19^	120,656 (AREDS); 5555 (KORA)	Zeiss FF series fundus camera; TOPCON TRC-NW5S 45° fundus camera	Normalization of color balance and local illumination by Gaussian filtering. Images resized to 512 x 512 to conform to neural network input dimensions	Features learned through neural network	ARMD severity

AI, artificial intelligence; ARMD, age-related macular degeneration; CLAHE, Contrast Limited Adaptive Histogram Equalization; RGB, red, green, blue; SIFT, Scale-Invariant Feature Transform; SURF, Speeded Up Robust Features; ARIA, automatic retinal image analysis; STARE, STructured Analysis of the REtina; AREDS, Age Related Eye Disease Study; OD, optic disc; ROI, region of interest; EUGENDA, The Euregio genetic database; KMC, Kasturba Medical College; RQA, recurrence quantification analysis; NIH, National Institutes of Health.

### 3.2 Preprocessing and feature extraction

In automatic detection, preprocessing is a commonly used step to enhance an image to better facilitate the extraction of features relating to objects of interest. The human eye distinguishes “features” of disease in an image (such as geographic atrophy and drusen), but AI algorithms need to extract “features” measured from the pixels pertaining to an object (i.e., drusen). In addition, a color fundus photograph typically contains a black border that needs either to be avoided or eliminated because these pixels will not be of any relevance. Retinal landmarks (e.g., the optical nerve boundary, blood vessels, and macula) may obstruct features of small objects, so their removal may further improve automatic detection by reducing sources of false targets for drusen detection. A color fundus photograph might also contain artifacts (e.g., from dust particles on the lens) and display areas of uneven illumination that preprocessing can eliminate. The type of preprocessing used in the studies included depended on the particular features used (Table 1).

Pixel values in imaging typically range from 0 (black) to 255 (white) per color channel (e.g., red, green, blue or hue, saturation, value). In color fundus photographs, drusen appear as small regions of bright pixels. Properties calculated from the image histogram (i.e., a plot of the number of pixels for each intensity value in the range and for each color channel) such as energy, entropy, and intensity have all been used as features for classifying whether regions in an image contain drusen or not. Contrast Limited Adaptive Histogram Equalization^48^ has been used^25^, ^42^, ^43^, ^61^, ^1^ to improve contrast in the image. This well-established technique involves flattening the image histogram of relative color intensities to make the whole image as similar as possible, ultimately enhancing histogram-based features. Two studies used a median filter, which is applied after removing the black border to smooth high-frequency noise, but at the cost of reducing contrast.^31^, ^47^ Grivensen and coworkers^20^ manually assigned individual pixels a probability that it is part of a drusen and automatically extracted their boundaries using intensity and contrast characteristics to then be used as features for training. Burlina and coworkers^7^ obtained training regions of background (no pathology) and testing masks for abnormal areas (candidate drusen) using standard image-processing techniques such as median filtering, morphological dilation, and thresholding. Garcia-Floriano and coworkers^18^ also used mathematical morphology to highlight drusen areas and healthy macular regions. Subsequently, features called Hu moments, a well-recognized tool for object recognition in computer science, were then calculated from each pixel.

After the preprocessing stage, it is necessary to select which features best perform as descriptors of the object of interest (i.e., drusen) within a classification scheme.

### 3.3 Feature selection

Feature selection, reported in 6 articles, is used to select a group from the extracted features or create variables that achieve the best classification performance. This process removes potentially irrelevant or confusing features and avoids model overfitting. In other words, it identifies salient features that can be used to distinguish disease images from healthy ones most effectively. Feature selection returns a numerical feature vector, which is the representation then used to train a classification algorithm (section 3.4).

Zheng^62^ used L2 loss function, an established FS technique. Their aim was to identify and filter the pixel intensity features that were produced by noise. The resulting list was then ranked, and the top features were selected to be used for disease/no-disease classification*.*

Garcia-Floriano and coworkers^18^ used a filter from a feature-selection software package.^21^ The filter uses correlation-based feature selection that evaluates the predictive capability of features and chooses subsets highly correlated to each class.^22^

To assess features that determine whether an image was dry or no ARMD, Mookiah and coworkers^42^, ^43^ used parametric and nonparametric tests (e.g., *t*-test and Wilcoxon ranking) to determine the top features, achieving the best one-versus-all classification for each class. With each ranked feature incrementally nested into the classification algorithm, they reported in one article^43^ a texture feature (from a Gabor filter) as the highest ranking. In their second article,^42^ the best feature was derived using the top energy features (entropy measures and their coefficients and averages) to compute an index for each image. The authors proposed the index value as a method for devising a threshold so that in a virtual clinic, the threshold would be used to determine dry ARMD from no ARMD.

In the study by Acharya and coworkers,^1^ feature selection was achieved combining a shortest-path algorithm, inspired by ants' behavior (ant colony optimization), with a genetic optimization algorithm, inspired by mutation and crossover operators in genetics (genetic algorithm). The overall aim was to classify dry ARMD and wet ARMD from no ARMD. The highest ranking energy and entropy features were selected according to analysis of variance to obtain a *P* value. The top 10 features (1 energy, 3 entropy, 6 other nonlinear) (Table 1) most statistically significant (P < 0.05) features were used for classification.

### 3.4 Classification

Classification uses the features selected to identify the model that best separates the data into the desired classes. A collection of images is typically separated into training and testing sets, of which the former is used to develop the model and the latter is used to test it. In the context of ARMD, this would test the model's ability to classify disease/no-disease or dry/wet ARMD. To evaluate the accuracy of the classifier, cross-validation is often performed.^52^ The algorithm performance is commonly reported in terms of statistics of measures, comparing the classifiers' decisions against those of one or more human experts (Table 2, Table 3, Table 4). Then, we describe the variety of classifications used in the studies included in this review.

**Table 2:** Included articles using ML for classification of disease/no disease

Reference	Images with disease (data set)	Images with no disease (data set)	Classifier	Reference standard	Performance
Hijazi et al^25^	86 (ARIA)	56 (ARIA)	Case-based reasoning (CBR)	Labels from ARIA project	ACC = 75%; SEN = 82.00%; SPEC = 65.00%
Burlina et al^7^	39 (private)	27 (private)	Constant false alarm rate (CFAR)	Graders from JHU Wilmer Eye Institute	SEN = 95%; SPEC = 96%; PPV (positive predictive value) = 97%; NPV (negative predictive value) = 92%
Zheng et al^61^	101 (ARIA); 59 (STARE)	60 (ARIA); 38 (STARE)	Naïve Bayes, SVM	Labels from data set	SPEC = 100%; SENS = 99.4%; ACC = 99.6%
Garcia-Floriano et al^18^	34 (STARE); 33 (RetinaGallery)	41 (STARE); 37 (RetinaGallery)	SVM	Labels from STARE and RetinaGallery	ACC = 92.1569%; precision = 0.904; recall = 0.922; F-measure = 0.921

ML, machine learning; SVM ,support-vector machine; ARIA, automatic retinal image analysis; STARTE, STructured Analysis of the REtina; AREDS, Age Related Eye Disease Study.

Performances reported as accuracy (ACC), sensitivity (SEN), and specificity (SPEC).

**Table 3:** Included articles using ML for classification of ARMD severity

Reference	Number of images in ARMD severity category	Classifier	Reference standard	ARMD category test	Performance
Kankanaballi et al^31^	EIPC: • 626 (category 1) • 89 (category 2) • 715 (category 3) • 715 (category 4) MIPC: • 626 (category 1) • 89 (category 2) • 1107 (category 3) • 950(category 4) MS: • 180 (category 1) • 13 (category 2) • 114 (category 3) • 78 (category 4)	Random forest	Expert grader	(1) {1 & 2} vs. {3 & 4} (2) {1 & 2} vs. {3} (3) {1} vs. {3} (4) {1} vs. {3 & 4}	EIPC: 95.4% (SPEC), 95.5% (SEN), 95.5% (ACC) MIPC: 91.6% (SPEC), 97.2% (SEN), 98.9% (ACC) MS: 98.4% (SPEC), 99.5% (SEN), 98.9% (ACC) EIPC: 96.1% (SPEC), 96.1% (SEN), 96.1% (ACC) MIPC: 95.7% (SPEC), 96.0% (SEN), 95.9% (ACC) EIPC: 98.6% (SPEC), 95.7% (SEN), 97.1% (ACC) MIPC: 96.3% (SPEC), 96.8% (SEN), 96.7% (ACC) EIPC: 96.0% (SPEC), 94.7% (SEN), 95.4% (ACC) MIPC: 95.4% (SPEC), 97.7% (SEN), 97.1% (ACC)
Grivensen et al^20^	Set A: • 17 observer 1, 20 observer 2 (no ARMD) • 13 observer 1, 9 observer 2 (early ARMD) • 22 observer 1, 23 observer 2 (intermediate ARMD) Set B: • 216 observer 1, 218 observer 2 (no ARMD) • 64 observer 1, 64 observer 2 (early ARMD) • 75 observer 1, 76 observer 2 (intermediate ARMD) Average number of drusen: • 130.4 ± 178.1 (observer 1), 198.5 ± 243.1 (observer 2) Average size of drusen (μm^2^): • 5,873 ± 10,027 (observer 1), 5115 ± 8257 (observer 2)	K-nearest neighbor; linear discriminant classifier; random forest	2 Observers	Drusen area: observer 1 vs. algorithm observer 2 vs. algorithm Interobserver Drusen diameter: observer 1 vs. algorithm observer 2 vs. algorithm Interobserver Risk assessment: observer 1 vs. algorithm observer 2 vs. algorithm	0.91 (ICC) 0.86 (ICC) 0.87 (ICC) 0.66 (ICC) 0.69 (ICC) 0.79 (ICC) 0.84 (observer SEN), 0.96 (observer SPEC), 0.948 (algorithm AUC), 0.765 (Kappa) 0.85 (observer SEN), 0.954 (observer SPEC), 0.954 (algorithm AUC), 0.760 (Kappa)
Phan et al^47^	Good quality: • 50 (category 1) • 43 (category 2) • 24 (category 3) • 22 (category 4) Poor quality: • 29 (category 1) • 36 (category 2) • 41 (category 3) • 34 (category 4)	SVM & random forest	2 graders	{1} vs. {2} vs. {3} vs. {4} {1 & 2} vs. {3} vs. {4} {1} vs. {2 & 3} vs. {4}	SVM: 62.7% (ACC) Random forest: 61.7% (ACC) SVM: 75.6% (ACC) Random forest: 74.2% (ACC) SVM: 72.4% (ACC) Random forest: 69.9% (ACC)

AREDS, Age Related Eye Disease Study; ARMD, age-related macular degeneration; EIPC, equal number of images; MIPC, maximum number of images per class; ML, machine learning; MS, manually selected images; SVM, support-vector machine.

Interclass correlation coefficient (ICC) was set at 95% confidence interval. Kappa scores measure interrater agreement. Performances reported as area under curve (AUC), sensitivity (SEN), specificity (SPEC), and accuracy (ACC). ARMD categories defined using AREDS categories^5^ or by in-house grading criteria (Cologne Image Reading Center and Laboratory [CIRCLE]).

**Table 4:** Included articles using ML for classification of wet/dry/no disease

Reference	Images with no disease (data set)	Images with ARMD (data set)	Classifier	Reference standard	Performance
Mookiah et al^43^	101 (ARIA) 36 (STARE) 270 (KMC)	60 (ARIA) 47 (STARE) 270 (KMC)	Naïve Bayes, K-nearest neighbors, decision tree, probabilistic neural network, SVM	Ophthalmologist group	ACC (ARIA) = 95.07% ACC (STARE) = 95.00% ACC (KMC) = 90.19%
Mookiah et al^42^	270 (KMC)	270 (KMC)	Naïve Bayes, K-nearest neighbors, probabilistic neural network, SVM	Ophthalmologist group	ACC = 93.70% SEN = 91.11% SPEC = 96.30%
Acharya et al^1^	404 (KMC)	517 Dry ARMD (KMC) 24 Wet ARMD (KMC)	SVM	Ophthalmologist group	ACC (PSO with SVM) = 85.12% SEN (PSO with SVM) = 87.2% SPEC (PSO with SVM) = 80%

ARMD, age-related macular degeneration; ML, machine learning; SVM, support-vector machine; PSO,  particle swarm optimization; ARIA, automatic retinal image analysis, STARE, STructured Analysis of the REtina; AREDS, Agre Related Eye Disease Study; KMC, Kasturba Medical College.

Performances reported as sensitivity (SEN), specificity (SPEC), and accuracy (ACC).

#### 3.4.1 Disease/no disease

Hijazi and coworkers^25^ proposed a case-based reasoning system to develop an automated screening tool to classify 144 color fundus photographs into ARMD or normal categories. Case-based reasoning is a problem-solving technique based on the observation of how humans use previous examples or information to solve new, but similar, problems. If a case-based reasoning system is given a new case, it will use the previous most similar cases in its case base to solve the problem. Each image histogram was conceptualized to a set of curves, called a time series, and used to generate a 2-step case-based reasoning classification. The first case consisted of enhanced green channel images, with the blood vessel pixels replaced with null values. The second case contained the same but with the further process of removing the optic disc. Histograms and their time series of a collection of unseen graded images were passed to the first case for comparison to the training images. An algorithm called dynamic time warping was used to measure the similarity between the histograms and time series of the testing and training images. If the unseen image was below a certain similarity measure, it was then passed to the second case for reassessment. The output is whether the input image is similar to either the learned time series of an ARMD image or a healthy image in the case base. A specificity of 82% was reported for the effectiveness of the classifier in identifying ARMD images, 65% specificity for the classifier identifying normal images, and 75% accuracy in classifying images as ARMD or normal (Table 2). This two-pass approach offered a system whereby isolation and segmentation of drusen was not required; however, removal of vessels and the optic disc was needed to improve the accuracy.

Constant false alarm rate detection is an adaptive algorithm that has been used to identify normal or intermediate ARMD in color fundus photographs. Constant false alarm rate is used in radar systems where true signal and noise signals need to be distinguished to determine origin. This returns a probability that the signal is not a false alarm. Burlina and coworkers^7^ adopted such a system on 66 color fundus photographs to separate ARMD from healthy images. Training and testing data were constructed from the masks obtained by preprocessing (normal retina tissue mask and edge/artifact mask). The constant false alarm rate detector was trained on the red, green, blue and hue, saturation, value color spaces of each mask, creating the signal that provides a feature for support-vector machine (SVM) classification. SVM classification is a form of ML based on regression in which data are projected to a much higher dimensional space to promote linear separability of the target classes. The ability of the classifier to determine whether the image contains interesting (i.e., potentially disease) changes was reported as having a 95% specificity and 95% sensitivity, with a positive predictive value of 97% and a negative predictive value of 92% (Table 2).

The same authors later reported image-mining techniques for disease/no-disease classification.^61^ In this method, images were represented as quadtrees, a form of hierarchical tree data representation, separated by their homogeny that is defined by similar pixel values. To extract features of the training image quadtrees, a mining algorithm was used to take features from the tree such as the pixel color similarity between parent and child nodes. This returned a set of features that were reduced using an SVM ranking method.^16^ To then classify the testing images, ML algorithms (Naïve Bayes and SVM) were used. Best detection was reported with SVM. This was then applied to new data to best predict which group the data should lie in. The authors reported 100% specificity, 99.4% sensitivity, and 99.6% accuracy. This system required blood vessel removal to improve its accuracy (Table 2).

Garcia-Floriano and coworkers^18^ used an SVM to classify 70 images into disease/no-disease categories. The proposed method was first evaluated on the entire data set with and without feature selection. They obtained an accuracy of 83.58% for both evaluations. The proposed method failed in certain images due to suboptimal image quality. Removal of poor-quality images and evaluation with feature selection improved accuracy to 92.16%.

#### 3.4.2 ARMD severity

Phan and coworkers^47^ attempted to classify ARMD severity according to their AREDS categories^5^ using visual words, also known as “bag of words.” The most salient features in the image were detected and their frequencies counted and binned in to a histogram. This forms a so-called vocabulary that can be used for automated detection of the same words in an unseen image. The authors used Speeded Up Robust Features to build the vocabulary from different color spaces (red, green, blue and a color space describing lightness, green-red, and blue-yellow, called L*a*b) of 279 images, including poor-quality images, to build the vocabulary. SVM and random forest classifiers were tested with and without feature-selection steps. They report the best performance for ARMD screening with SVM classifier (area under curve: 87.7%). For grading the classes of ARMD, they report {1} vs. {2} vs. {3} vs. {4} accuracy of 62.7%. Accuracy of 75.6% and 72.4% were obtained for {1&2} vs. {3} vs. {4} and for {1} vs. {2&3} vs. {4}, respectively (Table 3).

Kankanaballi and coworkers^31^ also used Speeded Up Robust Features along with a faster version called Scale-Invariant Feature Transform to extract local features in 2772 AREDS images. These features were taken from the L*a*b color space to generate a vocabulary for a visual words algorithm. They evaluated the performance of the algorithm to correctly classify images into AREDS categories^5^—(1) class {1&2} vs. {3 & 4}; (2) {1 vs. 2} vs. {3}; (3) {1} vs. {3}; (4) {1} vs. {3 & 4}—and experimented with 3 data set designs—a manually selected data set of good-quality images (denoted MS) and a set of automatically selected^44^ good-quality images, one where each class of AREDS category was as large as possible (denoted maximum number of images per class) and another where AREDS categories were kept equal (denoted equal number of images). They reported the highest accuracy for category 1 from MS images of 98.9% accuracy. For images automatically selected, the highest accuracies were 96.1% (category 2 equal number of images), 97.1% (category 3 equal number of images), and 97.1% (category 4 maximum number of images per class) (Table 3).

Grivensen and coworkers^20^ segmented drusen so that their location, area, and size could be quantified. The overall aim was to distinguish images of low-risk ARMD from high-risk ARMD. Two observers manually segmented 52 images to provide a reference set for evaluation of automated drusen quantification (set A) and graded 355 images to evaluate automated ARMD severity classification (set B). Candidate drusen extraction was achieved by convolving the green channel of the color fundus photographs with Gaussian filters and using their derivatives to train a classifier. The classifier used regression to determine the class of the data point and the pixels filter response, called K-nearest neighbors. The classifier can be used to assign a probability using the filter response of a previously unseen pixel that it belongs to a lesion. Therefore, neighboring pixels with high probabilities can be grouped into candidate drusen. At this stage, the authors segmented the optic nerve and blood vessels so that any candidate drusen overlapping these anatomical landmarks could be excluded. This produced a probability map of the image where a search-based optimization method (i.e., dynamic programming) was then used to solve the candidate borders. Subsequently, total drusen area and maximum drusen diameter were quantified and compared with measurements derived from the observers' manual annotations using intraclass correlation coefficients. Linear discriminant analysis was used to separate candidate drusen from true drusen by extracting over 100 features in different color space (Luv, Hue Saturation Intensity), intensity (red, green, blue contrasts), contextual (average, standard deviations of pixel probability inside/outside border), and shape (area, perimeter) information. Each image probability map was then binned according to candidate drusen size and used to train a random forest classifier. This builds a decision tree whereby the output is whether the image is from a low- or high-risk patient. The authors validated algorithm according to measurement agreeability between algorithm and two graders using intraclass correlation coefficient. They report intraclass correlation coefficients of drusen area and diameter measurements of 0.69 and highest area under curve of 0.954 of correct ARMD image classification (Table 3).

#### 3.4.3 Wet/dry/no disease

Using entropy measures as features from wavelet coefficients and from green channel CLACHE-enhanced images, detection of dry ARMD using SVM, Naïve Bayes, probabilistic neural networks, k-nearest neighbors, and decision trees was proposed by Mookiah and coworkers.^42^, ^43^ This system was trained and tested separately on three data sets (automatic retinal image analysis, STARE, and a private data set). The best performance was reported for an SVM classifier where Gabor, local pixel intensity changes, and entropy features ranked best. The highest performances were observed in automatic retinal image analysis and STARE, with an accuracy of correctly classifying between dry ARMD and normal of 95.7% and 95%, respectively.^43^ Statistical moments, energy, entropy, and Gini index features extracted from discrete wavelet transform (a well-known image denoising technique) also presented the best accuracy for SVM (93.70%).^41^ This system did not require prior segmentation of retinal landmarks and drusen, and the use of multiple classifiers provided a degree of discrimination ability of the extracted features (Table 4).

SVM was also reported to be the best performing classifier for pyramid histogram of gradients features extracted by the particle swarm optimization algorithm, used to detect wet ARMD and dry ARMD.^1^ In a private data set, 945 images were used for training and testing where the algorithm correctly identified the wet from dry from normal images with 85.12% accuracy. The number of wet ARMD images in the data set was imbalanced (21 dry to 1 wet). To compensate for this, synthetic samples was generated by oversampling of the minority class. This produced synthetic features to simulate pathology and balance the data set. This system did not require any retinal landmark or drusen segmentation steps (Table 4).

### 3.5 Deep learning

DL is a rapidly growing field where conventional ML feature extraction, training, and classifiers are replaced with multilayer neural networks capable of learning latent patterns in the data.^37^ Neural network architecture (i.e., the layers) are carefully designed and assembled for the task the network is to perform. Convolution, pooling, and fully connected layers are the basic building blocks for the most well-known class of neural networks, called convolutional neural networks. Convolutional neural networks are considered deep convolutional neural networks (DCNNs) when their architecture typically contains 10 or more convolutional layers. DCNNs require large amounts of often labeled data to train, that may not be available, especially in a health care setting. Various methods exist to increase data set size to use state-of-the-art DL techniques.

Tan and coworkers^55^ developed a 14-layer DCNN to classify images as disease/no disease and trained and tested on 1110 images (708 no disease and 402 disease). To increase the size of the data set, data augmentation was used. Images were flipped left, flipped down, and flipped left and downward to increase artificially the size of the data set. This produced four instances of each image used to train and test the DCNN. They validated the DCNN using 10-fold cross-validation reporting an average fold accuracy, sensitivity, and specificity of 95.45%, 96.43%, and 93.75%, respectively.

Pretrained networks also offer a solution when there are little data whereby networks already trained to solve a similar task can be reused (transfer learning). ImageNet is a large general (nonmedical) benchmark data set popularly used to develop DCNNs. Early layers of a DCNN learn lower level features such as edges and colors. The following layers learn higher level features and more image domain-specific features to classify the image. Transfer learning is based on the idea that these lower level features may generalize to images different from the training images. For instance, OverFeat is a pretrained network to detect and localize everyday objects within a nonmedical image.^51^ Burlina and coworkers^8^ assessed the efficacy of the pretrained DCNN in classification of ARMD using OverFeat. With the input of 5600 color fundus photographs from National Institutes of Health AREDS into the OverFeat network to classify against pairs of AREDS categories^5^ {1 & 2} vs. {3 & 4}; {1 & 2} vs. {3}; {1} and {1} vs. {3 & 4}, they reported a preliminary performance of 92% to 95% accuracy. The same experiment was performed in their later work^9^ to assess the use of these features to fine-tune an SVM classifier and compared the algorithms AREDS grades to a human grader. An input of 5664 images into the pretrained Overfeat network was used to obtain a feature vector. These features were then passed to an SVM classifier to classify ARMD images as before. They reported a similar performance between class 1 and class 4 and grader with less agreeability between class 2 and class 3, algorithm versus grader.

Ensemble learning is a method in which multiple models are combined into one predictive model. Grassman and coworkers^19^ trained six DCNNs from the ImageNet competition, independently,^11^, ^23^, ^36^, ^46^, ^53^, ^54^ to predict ARMD severity. Classes were defined as AREDS category (9 classes), late ARMD stages (3 classes), and ungradable image (1 class). The results from each DCNN were then used to train a random forest classifier to build a model ensemble. They trained and tested each DCNN and the ensemble on 120,656 color fundus photographs (86,770 training and 21,867 testing). Each individual DCNN achieved accuracies between 57.7% and 61.7%. By combining the DCNNs into an ensemble, the overall accuracy was increased to 92.1% for predicting each ARMD class. Grassman and coworkers^19^ also used an independent data set of 5555^6^ to evaluate their algorithm and achieved an accuracy of 34%. Misclassifications were color fundus photographs from healthy individuals incorrectly classified as neovascular ARMD. This was due to younger eyes in the KORA data set (<40 years old) demonstrating dominant macular reflexes, which was not observed in the training data (>55 years old). By restricting the analysis to fundus images of the eyes of individuals aged 55 years and older, they increased the performance to 50% accuracy for predicting ARMD severity according to their defined ARMD classes. When the algorithm was used to classify early or late ARMD, accuracy was improved to 84.2% and correctly classified 94.3% of healthy fundus images.

## 4 Discussion

Our search highlighted ML as the predominant technique for ARMD detection and classification, with most recent articles reporting DL techniques. The primary aim of drusen-related automated image analysis is to support decision-making in the clinic. Rather than detecting individual drusen, image-level classification was more common with the aim of computerizing ARMD screening and grading systems. Only a single article reported discrete drusen measurement and quantification.^20^ Manually outlining individual drusen to provide ground truth for algorithm training is very labor intensive and motivates the shortage of ML approaches to individual drusen segmentation. AREDS categories,^5^ class 1 and class 2 ARMD, are the most difficult to separate because grading relies on drusen counts and measurements that cannot be obtained automatically without the reference data. ML is particularly susceptible to this paradox because they are driven by examples that are assumed to be representative of the population. A newly obtained image may not be similar to any of the examples used to train the model, and therefore, it may fail to classify it. This effect of data variability was also observed in the study by Grassman and coworkers^19^ when the model was evaluated on an independent data set containing color fundus photographs with retinopathies not present in the training set and removal improved performance. This raises questions as to how ML would generalize to the clinic.

In terms of translating into the clinic, systems depending on segmentation of retinal landmarks^16^, ^20^, ^25^ would need reliable and robust detection and segmentation algorithms. Algorithms would also need to be robust to image quality. Comparably, Kankanaballi and coworkers^31^ and Phan and coworkers^47^ both used a visual words algorithm, but Kankanaballi et al^47^ included poor-quality images and achieved lower overall accuracies than Phan and coworkers who used a larger data set. In the study by Phan and coworkers,^47^ the algorithm is tested on data sets with a varying balance of images labeled in the ARED's categories, where highest accuracies are achieved for the more balanced data sets or category contains clear and expected differences between ARMD severities (class 1 vs. class {3 & 4}). This exemplifies how a classifier can be fine-tuned and stabilized by data set balance and image quality alone. In addition, Burlina et al^7^ used the only algorithm that explicitly states validation on African and Asian eyes, where because of high melanin content, images appear darker. This highlights that an algorithm for use in the clinic would also need to be robust to ethnicity.

Interestingly, the single article proposing a dry/wet classifier yielded good results^1^ even with synthetic data. Wet ARMD occurs when neovascularization occurs, with subsequent intraretinal fluid causing central vision loss. In the clinic, it is now standard practice to use cross-sectional optical coherence tomography for obtaining insight into intraretinal fluid levels. Presentation of wet ARMD involves a wide spectrum of changes in the retina from normal-looking retina to distorted bloody retina. This is a difficult classifier to train and may indicate why there is only a single report of an algorithm using ML to detect dry from wet ARMD. As DL is becoming a state-of-the-art technique for difficult classification problems, future studies using DL for classifying wet ARMD could yield better results. This would be valuable in the clinic because wet ARMD requires urgent care.

There is also a clear importance to assess algorithm performance against the expert grader if such systems are to be deployed in a clinical setting. The methods were evaluated on different data sets, which makes levels of performance difficult to compare between algorithms including, for example, variants in preprocessing, feature selection, and classification. Methods of preprocessing largely depend on the features that need to be enhanced, and the green channel is the most commonly reported input for drusen detection. Texture and color features are predominantly used for ARMD grading, which is reasonable considering that color distributions and texture in a diseased image may differ dramatically from those in a normal eye.

ML requires feature design and selection that increase in complexity as the data increase in variability. DL networks exploit underlying patterns that perform well when data complexity and variation increase. Given the variable nature of the human retina, such systems appear more promising for adoption in the clinic. As drusen edges are hard to define, DL may be able to learn subtle patterns within the data to aid in quantifying areas of drusen for detecting disease progression. DL algorithms are producing state-of-the-art results but come at a computational cost. Large amounts of data are required to train the data set, which still requires (some) validation from ground truth. Further development of such algorithms represents a growing and expanding interdisciplinary field for automatic disease detection.

The results of our search identified a number of articles reporting algorithms for detection of DR and glaucoma where drusen can also be present. Fundus imaging has also been used to derive biomarkers for systemic conditions, such as hypertension and diabetes.^40^ Recently, there are an increased number of reports linking ARMD to Alzheimer disease (AD). AD is diagnosed using medical history, psychiatric examination, brain imaging, and biomarkers in cerebrospinal fluid. Definitive classification requires neuropathological changes as seen on postmortem examination. Characteristic retinal changes have previously been identified in AD, such as a sparser retinal vascular network (inferring altered cerebral vasculature)^41^ and thinning of the retinal nerve fiber layer^56^ (a marker of axonal loss). A key component of AD-related deposits in the brain, amyloid β, is also found in drusen. Amyloid β is an aggregate-prone peptide family that aggressively targets neurons,^4^ and there are an increasing number of reports of amyloid plaques in the retina in patients with AD.^29^, ^35^, ^39^, ^59^ As the retina is anatomically, embryologically, and physiologically linked to the central nervous system, it is perhaps not surprising that these depositions may have implications to neurodegenerative disease of the brain. Indeed, the progression of drusen formation in the peripheral retina has been found to be more prevalent in patients with AD than in the age-matched control.^13^ These findings were in a small cohort but suggest a promising biomarker for disease-related plaque formation in the brain.

When ARMD progresses asymmetrically, patients risk remaining asymptomatic due to maintaining good visual acuity in their healthy eye. The resulting delay in presentation and treatment impacts visual prognosis.

For automated drusen assessment to be applied in the clinic, it must go beyond cross-sectional phenotyping and instead relate to real patient visual outcomes. Longitudinal studies will be required to determine if automated image grading, based on drusen detection, can accurately predict disease progression.

Future algorithms involving drusen detection should aim to provide useful quantification to aid screening for ARMD. A screening program should stratify patients according to optimal follow-up pathway. For automated drusen detection to contribute to the cost-effectiveness of a screening program for ARMD, it must separate individuals with drusen associated with normal aging from patients whose drusen load progresses and stratify patients with mild ARMD into those at low risk and at high risk of progression to severe ARMD. This would enable the ophthalmologist to select relevant patients for regular follow-up, thus improving the efficiency of patient care.

### 4.1 Method of literature search

Published studies were identified through systematic searches of EMBASE, PubMed, Web of Knowledge, Science Direct, ACM Digital Library, and IEEE Xplore. The search terms in the first instance included “*drusen*” and in combination with *“detection”* or *“classification”* or *“identification”* or *“segmentation”* or *“quantification”* or *“measurement”* or *“algorithm”*. Further filtering was conducted on the titles and abstracts based on whether they contain the phrase “*age-related macular degeneration”* or the abbreviation *“ARMD”*.

## Supplementary Data

Data Profile
